# Overcoming barriers to care for individuals with fetal alcohol spectrum disorder: challenges and solutions in New Mexico and beyond

**DOI:** 10.3389/fped.2025.1603765

**Published:** 2025-06-27

**Authors:** Aubrey C. Gibson, Juliet M. Velhagen, Talya Jaffe, Dina E. Hill, Marcia L. Moriarta, C. Fernando Valenzuela

**Affiliations:** ^1^ Department of Neurosciences, School of Medicine, University of New Mexico Health Sciences Center Albuquerque, New Mexico, NM, United States; ^2^ Department of Psychiatry and Behavioral Sciences, School of Medicine, University of New Mexico Health Sciences Center Albuquerque, New Mexico, NM, United States; ^3^ Department of Pediatrics, School of Medicine, University of New Mexico Health Sciences Center Albuquerque, New Mexico, NM, United States

**Keywords:** prenatal, alcohol, pregnancy, access, care

## Abstract

Fetal Alcohol Spectrum Disorder (FASD) encompasses a range of neurodevelopmental impairments caused by prenatal alcohol exposure, affecting cognitive, behavioral, and social functioning. Despite its high prevalence, FASD remains underdiagnosed, particularly in regions like New Mexico (NM), where high rates of alcohol use, poverty, and unplanned pregnancies exacerbate the burden of the disorder. Individuals with FASD often face significant challenges in adaptive functioning, education, and social integration, with many experiencing adverse childhood events that compound developmental difficulties. Access to appropriate healthcare and social services is hindered by diagnostic complexity, stigma, and exclusion from disability benefits. Limited awareness among healthcare providers, educators, and social workers further impedes early intervention, leading to increased risks of poor academic performance, unemployment, homelessness, and criminal justice involvement. Specialized FASD clinics play a critical role in diagnosis and support, but remain insufficient to meet the needs of affected individuals. Expanding education, advocacy, and tailored support services is essential to addressing these gaps. By enhancing awareness, integrating FASD-specific disability benefits, and strengthening community-based programs, long-term outcomes for individuals with FASD can be significantly improved.

## Introduction

Fetal Alcohol Spectrum Disorder (FASD) includes a group of deficits that occur in individuals who were exposed to alcohol *in utero* (1). It is the most common teratogen-caused neurodevelopmental disorder, with an estimated prevalence of 3%–10% in the United States (2, 3). Deficits can range in severity and have long-lasting detrimental effects on an individual's social and cognitive abilities. Despite the high prevalence of these conditions, resources and social support, including government aid, social programs, and educational awareness, are severely lacking across the nation. This lack of resources disproportionately affects people in New Mexico (NM) and other states with high poverty rates, where the elevated prevalence of alcohol use in women of reproductive age is of particular concern. NM has a high burden of alcohol use, with the highest rate of alcohol-related deaths per capita (4), and 16.9% of females ages 18–44 who reported binge drinking from 2021 to 2022 (5). In one survey, 5% of women in NM reported drinking during the third trimester of pregnancy (6). Additionally, the rate of unplanned pregnancy in NM was one of the highest in the nation in 2018 (43.2%) (7).

**Figure 1:**
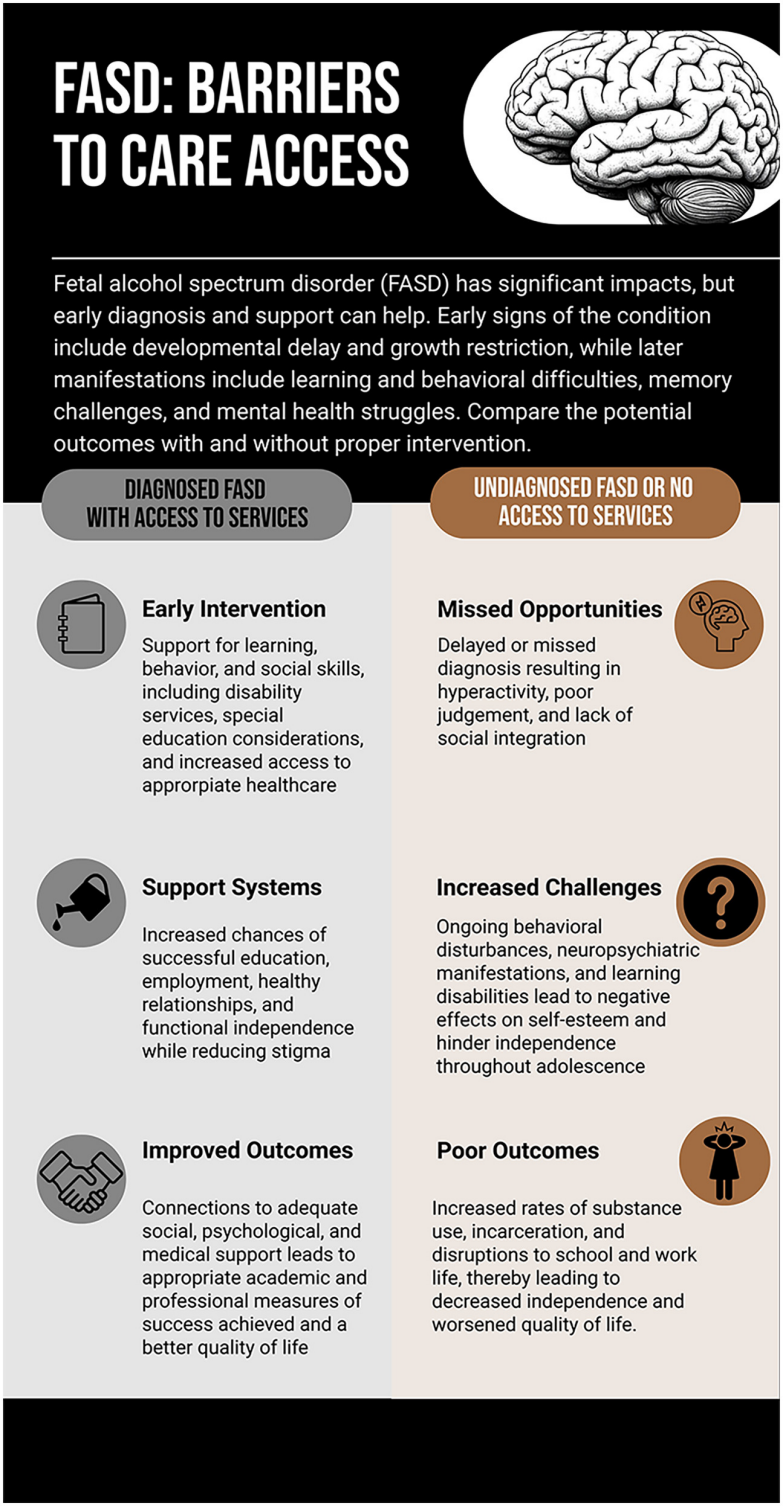
Infographic titled “FASD: Barriers to Care Access” illustrating outcomes for individuals with fetal alcohol spectrum disorder (FASD). The left side shows diagnosed individuals with access to services, who experience early intervention, supportive resources, and improved life outcomes. The right side depicts undiagnosed individuals without service access, facing missed interventions, greater behavioral challenges, and poorer outcomes such as substance use and reduced quality of life. Created using Piktochart (https://create.piktochart.com/output/fdb3719f6b7f-fetal-alcohol-spectrum-disorder-when-it-is-diagnosed-and-pro).

### Neurobehavioral consequences of prenatal alcohol exposure

FASD can affect various developmental areas, including general intelligence, motor function, attention and activity levels, language development, visual perception, learning and memory, adaptive functioning, and executive functioning (8, 9). Individuals with FASD can display maladaptive behaviors in childhood, which in turn can result in adverse life outcomes, including disrupted school experiences, trouble with the law, confinement, and substance use problems (10). Studies comparing individuals with FASD to IQ-matched individuals with another specific learning disorder showed that the FASD group consistently displayed lower adaptive functioning, which can manifest as difficulty with communication, socialization, and acquiring skills throughout life. These difficulties can become more severe with age if not recognized and addressed promptly (11, 12).

Although the inciting event for these impairments is exposure to alcohol *in utero*, several environmental and social factors have been identified that can perpetuate increased challenges throughout life for individuals with FASD. Many children with FASD have also been shown to experience adverse childhood events (ACEs), such as early maternal death, physical or emotional abuse, substance use in the home, being removed from their home, and having transient living environments, all of which may contribute to increased challenges for these children (10). Individuals with FASD may already be in a position of adversity, as many mothers of children with FASD live below the poverty line, have experienced physical or sexual abuse, or have mental health diagnoses (13). The combination of these factors means that children with FASD are often additionally exposed to ACEs, which may have compounding negative social and behavioral effects on a child, especially in the context of the neurodevelopmental effects of prenatal alcohol exposure.

Underdiagnosis and delays in diagnosis of FASD interfere with accessing proper healthcare and the social support that may help to overcome many difficulties experienced by individuals with FASD. Research suggests that the longer a child goes without an official FASD diagnosis, the higher the chance of deleterious consequences (10). Moreover, children with FASD frequently have other comorbid conditions that affect cognitive and social functioning, such as attention deficit-hyperactivity disorder and other mental health conditions, further convoluting the approach to diagnosis and treatment (13). Conversely, living in a stable and nurturing home and receiving a diagnosis before six years of age have been shown to protect against the adverse outcomes discussed above (2, 10). Eliminating some environmental factors that could hinder healthy development allows children with FASD to achieve their full potential through understanding their diagnosis and obtaining proper assistance.

### Care needs for individuals with FASD

Individuals with FASD face numerous challenges that can significantly impact their ability to function independently and thrive. Developmental delays, including difficulties with speech, motor skills, and cognition, are common, as are learning disorders and attention deficits (3). Addressing these issues often requires specialized education, therapy, and tailored support. Behavioral and emotional difficulties, such as poor impulse control and frustration management, further highlight the need for mental health services like FASD-informed counseling and behavioral therapy. Additionally, physical health concerns, including coordination problems and congenital abnormalities, may necessitate medical and therapeutic interventions (14).

Social and adaptive skills deficits also pose barriers for individuals with FASD, often making it difficult to navigate relationships or meet societal expectations. Programs focused on social skills training and community integration are essential for building confidence and fostering independence. Without targeted support, individuals with FASD are at a higher risk for secondary challenges, such as unemployment, homelessness, substance abuse, or legal issues. Preventative services, including job coaching, life skills training, and vocational programs, are critical in mitigating these risks and promoting long-term success (15).

FASD is a lifelong condition, meaning many individuals may benefit from ongoing support throughout adulthood (16). Services like case management, financial assistance, and housing support are vital for stability for some individuals. Families and caregivers may also benefit from education, respite care, and advocacy resources to better manage the unique needs of individuals with FASD. Furthermore, legal protections under the American Disabilities Act and Section 504 ensure access to necessary accommodations. Early and sustained access to tailored services enhances quality of life and raises community awareness, fostering greater inclusion and understanding (10).

### Barriers to care

Individuals with FASD face many adversities when it comes to accessing care, from difficulty with receiving a diagnosis, exclusion of the diagnosis from disability eligibility, lack of education relating to FASD in healthcare and school settings, and lack of social work services equipped to support these individuals (Figure 1).

#### Lack of awareness and recognition

FASD diagnosis is complex, requiring the identification of morphological and/or functional anomalies. Due to the nuanced diagnostic criteria, FASD diagnoses are most accurately made by specialists such as developmental pediatricians or neuropsychologists. However, in regions like NM, where poverty rates are high and many individuals reside in rural areas, assessing the need for services and allocating resources effectively poses significant challenges. As a result, many individuals with FASD remain undiagnosed and lack access to critical support (14).

FASD has not received the same research or social support as other neurodevelopmental disorders. The Diagnostic and Statistical Manual of Mental Disorders (5th edition) (DSM-5) category “Neurodevelopmental Disorder Associated with Prenatal Alcohol Exposure” (ND-PAE) only partially addresses FASD without considering the full spectrum of anomalies associated with this condition (17). The diagnostic criteria for these conditions are challenging (8), relying on healthcare providers’ identification of specific anomalies relating to growth, central nervous system function, and facial features (11). In most cases, maternal alcohol use cannot be confirmed, making the diagnosis more difficult, particularly in cases that lack the characteristic facial anomalies (8, 18). Even when a diagnosis is made, some providers who specialize in treating FASD may not accept Medicaid, a common form of insurance for affected families, adding another barrier to accessing care (2).

Stigma, judgment, and misconceptions compound these challenges (19–21). The stigma surrounding prenatal alcohol exposure discourages families from seeking or disclosing the need for services, fearing judgment or blame for the child's difficulties. Misdiagnosis or failure to diagnose not only delays critical interventions but also exposes individuals with FASD to additional risks, such as mental health disorders or social dysfunction. Moreover, some providers perceive individuals with FASD as “unfixable”, leading to inadequate efforts to provide meaningful support. Without proper recognition and intervention, children with FASD may face heightened risks of secondary challenges, such as poor educational outcomes, unemployment, and involvement with the criminal justice system (22). Addressing these systemic and social barriers is essential for improving outcomes and ensuring equitable access to care.

#### Exclusion from disability services and limited social support

In NM and many other states, individuals with FASD face significant barriers to accessing disability services and social support despite the long-lasting challenges associated with the condition (2, 23). FASD is not explicitly included in the eligibility criteria for developmental disability benefits under the state's waiver program. While children with intellectual disabilities resulting from FASD may qualify if their IQ and adaptive functioning scores are below specific thresholds, many individuals with Alcohol-Related Neurodevelopmental Disorder fail to meet the stringent requirements despite having significant limitations at multiple levels. This exclusion leaves many without access to the critical services they need for education, therapy, and daily living support.

The lack of awareness and preparedness among professionals further exacerbates the issue. Research highlights that over 70% of social workers do not feel equipped to manage FASD cases, reducing the likelihood of referrals to appropriate services (13). Educators may misinterpret the behaviors of children with FASD as intentional disruptions rather than symptoms of their condition, resulting in punitive measures instead of tailored educational support (2, 9, 23). Furthermore, the stigma surrounding prenatal alcohol exposure discourages families from seeking help, while a lack of public education campaigns perpetuates misconceptions about the condition.

Access to services is also hindered by systemic challenges, such as the high cost of care, insurance limitations, and income-based restrictions that exclude middle-income families. The lengthy, complex application processes for services like Social Security Income or Medicaid and frequent denials due to insufficient documentation or lack of diagnosis create additional barriers. For adults with FASD, the transition out of school-based support often means losing access to any form of assistance, as typically, there are no dedicated clinics or services for adults with FASD. To address these gaps, community programs must expand their scope to meet the needs of FASD families, and education efforts must increase awareness among teachers, healthcare providers, and social workers to improve diagnosis, access, and long-term outcomes.

Families in rural and remote areas of NM and other states face additional obstacles in accessing services for FASD. The scarcity of diagnostic centers and specialists in these regions often requires long travel distances, and a lack of reliable transportation can make attending appointments nearly impossible. Cultural and language barriers may compound these challenges, as services may not be tailored to the diverse populations they aim to support, leaving families from culturally distinct backgrounds underserved. Moreover, fear of potential involvement with child protective services can discourage families from seeking help, leaving many individuals with FASD without the critical support they need to thrive (19, 20). Addressing these barriers requires a concerted effort to expand rural access, improve cultural sensitivity in service delivery, and foster trust with underserved communities.

### The importance of specialized FASD clinics and alternative approaches in overcoming these barriers

Specialized FASD clinics play a critical role in addressing the diagnostic and treatment challenges faced by individuals with prenatal alcohol exposure. NM and several other states (e.g., Alaska, California, Florida, Georgia, Minnesota, Michigan, Nebraska, New York, South Dakota, and Washington) have established clinics dedicated to evaluating and supporting individuals exposed to substances during pregnancy, including alcohol. In NM, the FASD clinic at the Center for Development and Disability at the University of New Mexico School of Medicine employs an interdisciplinary team, including a pediatric neuropsychologist, a pediatrician, social workers, occupational therapists, and speech therapists. This clinic provides comprehensive diagnostic evaluations using Institute of Medicine criteria (18) and offers limited services to support patients and their families.

Despite these efforts, significant gaps remain. Most interventions for children with FASD are managed through the educational system, leaving care fragmented and dependent on individual school resources. Individuals in NM often face delays in obtaining diagnoses, with appointment wait times extending to six months or longer, compounded by challenges in retrieving records from outside facilities. Once individuals with FASD transition into adulthood, either by graduating from high school or aging out of foster care, their access to support diminishes drastically. With no clinics or organizations specifically focused on adults with FASD in New Mexico—and only a few available nationwide—services often cease at the very time individuals need assistance navigating the workforce and achieving independence.

Addressing these challenges requires expanding specialized FASD clinics to serve individuals across the lifespan and integrating alternative approaches, such as community-based programs and telehealth services, to bridge gaps in care and improve long-term outcomes for individuals with FASD. Despite various setbacks, additional support measures are available to help mitigate adverse effects. Professional organizations, such as FASD United at a national level and Families ASAP in NM, and local community workers provide essential reinforcements for families, offering assistance in managing a diagnosis of FASD. This may include attending meetings for Individualized Education Programs, advocating for tailored educational services, and providing critical education about FASD to caregivers and educators. The UNM FASD clinic also collaborates closely with the foster care system, receiving referrals from across the state, ensuring that children in state care have an opportunity for diagnosis and support.

State-level interventions, particularly in education and awareness, have the potential to make the most significant difference for patients with FASD in NM and across the country. Expanding knowledge about FASD among teachers, caregivers, counselors, coaches, and other adults who interact with children is critical to providing timely and effective care and support. Greater awareness of FASD signs and symptoms could lead to earlier diagnoses, improved access to specialized services, and better long-term outcomes for affected individuals. While NM has numerous community programs to support underserved populations, these initiatives must be adapted to meet the unique needs of individuals with FASD and their families. By refining existing resources and fostering a more informed network of caregivers and service providers, NM and other states can reduce diagnostic delays, enhance support systems, and help individuals with FASD achieve their full potential.

## Conclusions

Addressing the challenges faced by individuals with FASD requires a multifaceted approach integrating education, awareness, and improved access to resources. The pervasive lack of knowledge about FASD within both the community and healthcare systems has perpetuated delayed diagnoses and limited access to essential services. Expanding educational initiatives for healthcare providers, educators, and community members on recognizing and managing FASD is critical to reducing stigma and fostering earlier interventions. Increasing the availability of social, educational, and disability resources, as well as advocating for FASD-specific eligibility for disability benefits, could significantly improve the quality of life for affected individuals and their families. In states like NM, where the burden of FASD is exceptionally high, leveraging existing community programs and systems to address the unique needs of this population presents a clear opportunity for meaningful change. By implementing these strategies and fostering collaboration among health, education, and social services, the potential for individuals with FASD to lead more fulfilling and stable lives can be realized.

## Data Availability

The original contributions presented in the study are included in the article/Supplementary Material, further inquiries can be directed to the corresponding author.
